# Hepatitis B vaccine uptake, completion, and associated factors among university students in Tanzania: a mixed method study at KCMC University, Moshi, Tanzania

**DOI:** 10.1186/s12879-026-13501-5

**Published:** 2026-05-04

**Authors:** Emmanuel Thomas Issangya, Dhahiri Mnzava, Barnabas Gabriel, Basiliana Emidi, Geofrey Nimrod Sigalla, Debora Charles Kajeguka

**Affiliations:** 1 Department of Microbiology and Immunology, KCMC University, Moshi, Tanzania; 2 National AIDS, STI and Hepatitis Control, Dodoma, Tanzania; 3 National Institute for Medical Research, Dodoma, Tanzania; 4 Zanzibar Health Research Institute (ZAHRI), Binguni, Zanzibar, Tanzania

**Keywords:** Hepatitis B, Vaccine uptake, Vaccine completion, University students, Moshi, Tanzania

## Abstract

**Background:**

Hepatitis B Virus (HBV) infection remains a significant global public health concern, particularly in sub-Saharan Africa, where prevalence ranges between 6% and 8% among the general population. HBV is a major cause of chronic liver disease, cirrhosis, and hepatocellular carcinoma. Vaccination against HBV is highly effective in preventing infection; however, uptake and completion of the vaccine series remain suboptimal in many countries, including Tanzania. This study aimed to determine the uptake and completion of HBV vaccination and associated factors among students at KCMC University, Moshi, Tanzania.

**Methods:**

A mixed-methods study was conducted among 284 students at KCMC University, Tanzania. Data were collected using a pretested structured questionnaire, which captured information on socio-demographic characteristics, knowledge of hepatitis B virus infection and vaccination, and perceptions toward HBV vaccination. Data were analysed using descriptive and inferential statistics. A *p*-value of < 0.05 was considered statistically significant. Qualitative data were transcribed, thematically analysed, and subjected to content analysis to identify recurring themes and sub-themes.

**Results:**

A total of 284 participants were enrolled. with a mean age of 22.9 ± 3.9 years, and 175 (61.6%) were female. Vaccine uptake, defined as receiving at least one dose of the hepatitis B vaccine was 113 (39.8%), and only 68 (23.9%) completed all three doses. Uptake of vaccination was significantly associated with year of study *(p* < 0.05), program level (*p* = 0.008), knowledge of hepatitis B (*p* = 0.001), and awareness of mass vaccination campaigns (*p* = 0.003). Completion of the vaccine series was higher among bachelor’s students (AOR = 1.8, 95% CI: 0.09–0.57, *p* = 0.001), participants who received training on hepatitis infection (AOR = 2.3, 95% CI: 1.2–7.6, *p* = 0.021), those knowledgeable about hepatitis B infection and prevention (AOR = 5.34, 95% CI: 2.2–4.7, *p* = 0.001), and who were aware of mass vaccination campaigns (AOR = 0.22, 95% CI: 0.12–0.27, *p* = 0.001).

**Conclusion:**

Hepatitis B vaccine uptake and completion among students remain low, indicating gaps in protection against HBV infection. Uptake was significantly associated with year of study, program level, knowledge of hepatitis B, and awareness of vaccination campaigns. Completion was linked to bachelor’s level, hepatitis training, and knowledge. Strengthening targeted education, training, and vaccination campaigns is recommended.

**Supplementary Information:**

The online version contains supplementary material available at 10.1186/s12879-026-13501-5.

## Background

Hepatitis B virus is a liver infection caused by the HBV virus and represents one of the major global public health challenges [1, 2]. According to the World Health Organization (WHO), more than 296 million people worldwide are living with chronic HBV infection, and over 820,000 deaths occur annually due to complications such as cirrhosis and hepatocellular carcinoma [3]. Hepatitis B virus (HBV) infection remains a major global public health concern, particularly in sub-Saharan Africa, where it contributes significantly to chronic liver disease and related complications, with prevalence estimates ranging between 6% and 8% in the general population [3, 4].

Despite the availability of a safe and effective vaccine, uptake and completion of the hepatitis B vaccine remain suboptimal among university students in many low- and middle-income countries, including Tanzania [5]. The virus is primarily transmitted through exposure to infected body fluids, including blood, semen, and other bodily secretions [6]. In high and intermediate endemic regions such as Tanzania, perinatal transmission particularly during or after delivery is more common than in utero transmission, contributing significantly to the burden of chronic infection [3]. Tanzania continues to experience a significant burden of HBV infection, with prevalence estimates ranging from 3.8% to 8% among the general population [7, 8].Sexual transmission of HBV can also occur, particularly among unvaccinated individuals with multiple sexual partners, men who have sex with men, people who inject drugs, healthcare workers, and patients undergoing regular blood transfusions or haemodialysis [9]. University students, especially those in health-related programs, are at increased risk of exposure due to clinical training and laboratory activities [10]. However, studies have reported incomplete vaccination schedules among students, largely due to inadequate awareness, misconceptions about vaccine safety, and logistical barriers such as limited access to vaccination services [11]. This gap between vaccine availability and completion highlights the need to assess uptake and associated factors among university students to inform targeted interventions.

The World Health Organization recognizes HBV as a major public health threat and recommends universal vaccination as a primary preventive measure. The HBV vaccine is highly effective, offering 90–95% protection against the infection when administered in a complete series [12]. Despite the availability of the vaccine, uptake remains suboptimal in many regions, including Tanzania. In some healthcare settings, vaccination coverage among healthcare workers has been reported as low as 33% indicating a significant gap in preventive healthcare practices [13].

The risk of HBV exposure among university students, particularly those in healthcare-related programs, is elevated due to potential occupational hazards such as needle-stick injuries and exposure to blood and bodily fluids during clinical training [14]. However, studies focusing on HBV vaccination uptake and completion among university students in Tanzania are limited. Existing research primarily addresses healthcare workers, with findings suggesting that factors such as vaccine unavailability, time constraints, and lack of awareness contribute to low vaccination rates [15]. These insights underscore the necessity for targeted interventions to enhance HBV vaccination among students in healthcare programs. Addressing this gap, the current study aimed to assess the prevalence of HBV vaccination uptake and completion among students at KCMC University and to identify factors influencing these outcomes.

## Methods and data collection

### Study design and setting

A mixed-methods study was carried out at KCMC University, located in Moshi Municipality, Kilimanjaro Region, northern Tanzania, from May to August 2025 to determine the uptake and completion rates of hepatitis B vaccination and to explore associated factors. The quantitative part employed a cross-sectional design to estimate vaccination coverage and completion levels, while the qualitative part used a descriptive design involving Key Informant Interviews (KIIs) conducted in parallel to obtain an in-depth understanding of the factors influencing students’ vaccination uptake and completion.

### Study population and eligibility

The study population consisted of diploma and undergraduate students enrolled at KCMC University during the study period. Eligible participants included all students who were registered at the university and provided informed consent to participate in the study. Students who were severely illness (defined as any acute or chronic condition requiring hospitalization or rendering the student physically/mentally unable to complete the questionnaire at the time of data collection) at the time of data collection or unwilling to participate were excluded.

### Sample size and sampling technique

A total of 284 students were recruited, based on the Kish Leslie formula for prevalence studies, assuming a 18% prevalence of HBV vaccination uptake [15], a 5% margin of error, and a 95% confidence level, with an additional 10% to account for non-response. Stratified random sampling was employed to ensure adequate representation across different academic levels and faculties at KCMC University. Academic levels were categorized into diploma and undergraduate (bachelor’s) programs, while faculties included the various academic disciplines within the university, such as medicine, nursing, and allied health sciences. The sampling process began by obtaining a complete list of registered students from the university registry. Students were then stratified based on their level of study and further categorized by faculty to ensure proportional representation across disciplines. The required sample size was proportionally allocated to each stratum according to the number of eligible students within each category. Within each stratum, eligible students were assigned unique identification numbers, and simple random sampling was applied using a random number generator to select participants. In cases of non-response or refusal, additional participants were selected using the same procedure until the required sample size was achieved.

For the qualitative component, participants were purposively selected to ensure inclusion of individuals with relevant experiences and knowledge on hepatitis B vaccination uptake, allowing for in-depth exploration of perceptions and barriers. Data were collected through Key Informant Interviews (KIIs) and Focus Group Discussions (FGDs) until thematic saturation was reached. Data collection continued until no new themes, ideas, or relevant information emerged from consecutive interviews or discussions. Saturation was determined when additional KIIs and FGDs no longer contributed new insights relevant to the study objectives, indicating that sufficient information had been obtained to adequately address the research questions. A total of 13 KIIs and 3 FGDs were conducted. Each interview lasted approximately 30–60 min, while FGDs lasted 60–90 min.Homogeneity in FGDs was maintained by grouping participants based on similar academic levels to minimize variability in experiences and ensure consistency of discussion. The interviews and FGDs were conducted by trained research assistants, while the principal investigator supervised the data collection process to ensure adherence to the study protocol, ethical standards, and data quality assurance.

### Data collection, tools, and procedures

A semi-structured, self-administered questionnaire consisting of seven sections was used for quantitative data collection. The questionnaire was developed and subsequently pretested to ensure clarity, validity, and reliability of the items. Necessary revisions were made based on feedback from the pilot testing. The finalized tool was programmed into Kobo Toolbox for digital data collection and deployed on Kobo Collector mobile devices (Supplementary File 1). Trained interviewers administered the tool to gather information on socio-demographic characteristics, knowledge, and perceptions regarding Hepatitis B vaccination.

The first section captured socio-demographic characteristics, while the second assessed participants’ exposure to HBV and associated risk factors. Third section assessed awareness of HBV measured using a single question asking whether participants had previously heard of the infection. Fourth one assessed knowledge focusing on transmission, prevention, and treatment, to gauge participants’ understanding of the disease [9]. The fifth section explored HBV vaccination status by asking participants if they had been vaccinated and the number of doses received. Participants who had completed three doses were classified as vaccinated, while those with fewer doses or none were considered unvaccinated. Sixth section captured, perceptions toward HBV infection and vaccination were evaluated using the six constructs of the Health Belief Model (HBM): perceived severity, susceptibility, benefits, barriers, self-efficacy, and cues to action. Each construct included multiple statements framed as questions [16]. The final section collected additional information relevant to HBV prevention and vaccination practices.

In addition to the quantitative collection, qualitative data were collected through semi-structured Key Informant Interviews (KIIs) to explore factors influencing Hepatitis B vaccination uptake and completion. A KII guide was developed based on thematic areas aligned with the Health Belief Model, including knowledge and awareness, perceived susceptibility and risk, motivators and barriers to completion, influence of peers and family, and accessibility and convenience of vaccination services (Supplementary File 2).

### Pretesting and training

A pilot study involving 20 students from a similar academic institution, excluded from the final sample, was conducted to assess the tool’s clarity and appropriateness. Insights gained from the pilot exercise guided refinement of the instrument. Data collectors were adequately trained on study protocols, ethical requirements, and uniform questionnaire administration to enhance reliability and ensure high-quality data collection.

### Data analysis

Completed questionnaires were checked for completeness, coded, and entered into a computer database. Data coding was performed by two trained research assistants under the supervision of the principal investigator. A pre-defined coding scheme was developed prior to data entry to ensure consistency and reduce errors. Quantitative data were numerically coded and entered into IBM SPSS Statistics for analysis. Qualitative data were transcribed verbatim, translated into English where necessary, and manually coded using a thematic coding framework. The coded qualitative data were then analyzed thematically to identify emerging patterns and themes. Descriptive statistics (frequencies, percentages, means, and standard deviations) summarized participant characteristics and vaccination status. Vaccination uptake was defined as the proportion of participants who reported receiving at least one dose of the hepatitis B vaccine at the time of the study, including those who had received one, two, or all three doses. Vaccine completion was defined as receiving all three recommended doses of the hepatitis B vaccine according to standard vaccination guidelines recommended by the World Health Organization. Knowledge of hepatitis B infection was assessed using multiple items covering transmission routes, prevention methods, treatment, and complications. Correct responses were scored 1, while incorrect or “Not sure” responses were scored 0. A composite knowledge score was calculated and converted into a percentage. Participants scoring ≥ 70% were classified as having good knowledge, while those scoring < 70% were classified as having poor knowledge [17]. Awareness, vaccination status, and perception constructs were similarly measured, with perception items rated on a five-point Likert scale (1 = strongly disagree, 5 = strongly agree). Mean construct scores were computed for analysis.

Univariate logistic regression was performed for each independent variable to explore relationships between vaccination outcomes and independent variables. Variables that demonstrated statistical significance (*p* < 0.05) were subsequently entered into multivariable logistic regression models to determine independent predictors of vaccination uptake and completion. The strength of associations was expressed using odds ratios (ORs) together with their corresponding 95% confidence intervals (CIs). Qualitative data were analysed thematically. Transcripts were coded manually and grouped into themes reflecting perceptions, barriers, and facilitators of HBV vaccination. The findings from both quantitative and qualitative components were integrated during the interpretation phase using a triangulation approach. This method was employed to enhance the validity and depth of the findings by comparing and complementing results from both datasets. Integrating the two components provided a more comprehensive understanding of the determinants influencing hepatitis B vaccination uptake and completion among students.

## Results

### Demographic characteristics of participants

A total of 284 participants were involved in the study. The majority 222 (78.2%) were aged between 17 and 23 years, with a mean age (Mean of ± SD) (22.9 ± 3.9). In terms of gender, there were more females 175 (61.6%) compared to male participants. Regarding marital status, the vast majority were single 261 (91.9%). Meanwhile, participants were distributed across different years of study, with the largest group in Year 3 with 92 (32.4%). Most participants were pursuing Diploma programs 172 (60.6%). The majority identified as Christians 201 (71.3%). Just over half of the participants 146 (51.8%) had attended training on hepatitis infection and prevention. Addittionally,174 (61.3%) did not receive any dose of HBV vaccine (Table 1).

**Table 1:** Participants’ demographic characteristics (*n* = 284)

Variable	Response (*n*)	Percentage (%)
**Age**		
17–23	53	18.7
24–30	222	78.2
> 31	9	3.2
**Sex**		
Male	109	38.4
Female	175	61.6
**Marital status**		
Married	19	6.7
Single	261	91.9
Other	4	1.4
**Year of study**		
Year 1	67	23.6
Year 2	84	29.6
Year 3	92	32.4
Year 4	41	14.4
**Program level**		
Diploma	172	60.6
Bachelor	112	39.4
**Religion**		
Christian	204	71.8
Muslim	74	26.1
Other	6	2.1
**Have you attended training on Hepatitis infection and prevention (** ***n*** ** = 283)**		
Yes	146	51.8
No	137	48.2
**Have you taken vaccine**		
Yes	113	39.8
No	171	60.2
**Number of doses received**		
0 doses	174	61.3
1 dose	21	7.4
2 doses	21	7.4
3 doses	68	23.9

### Prevalence of vaccine uptake and completion

**Fig. 1:**
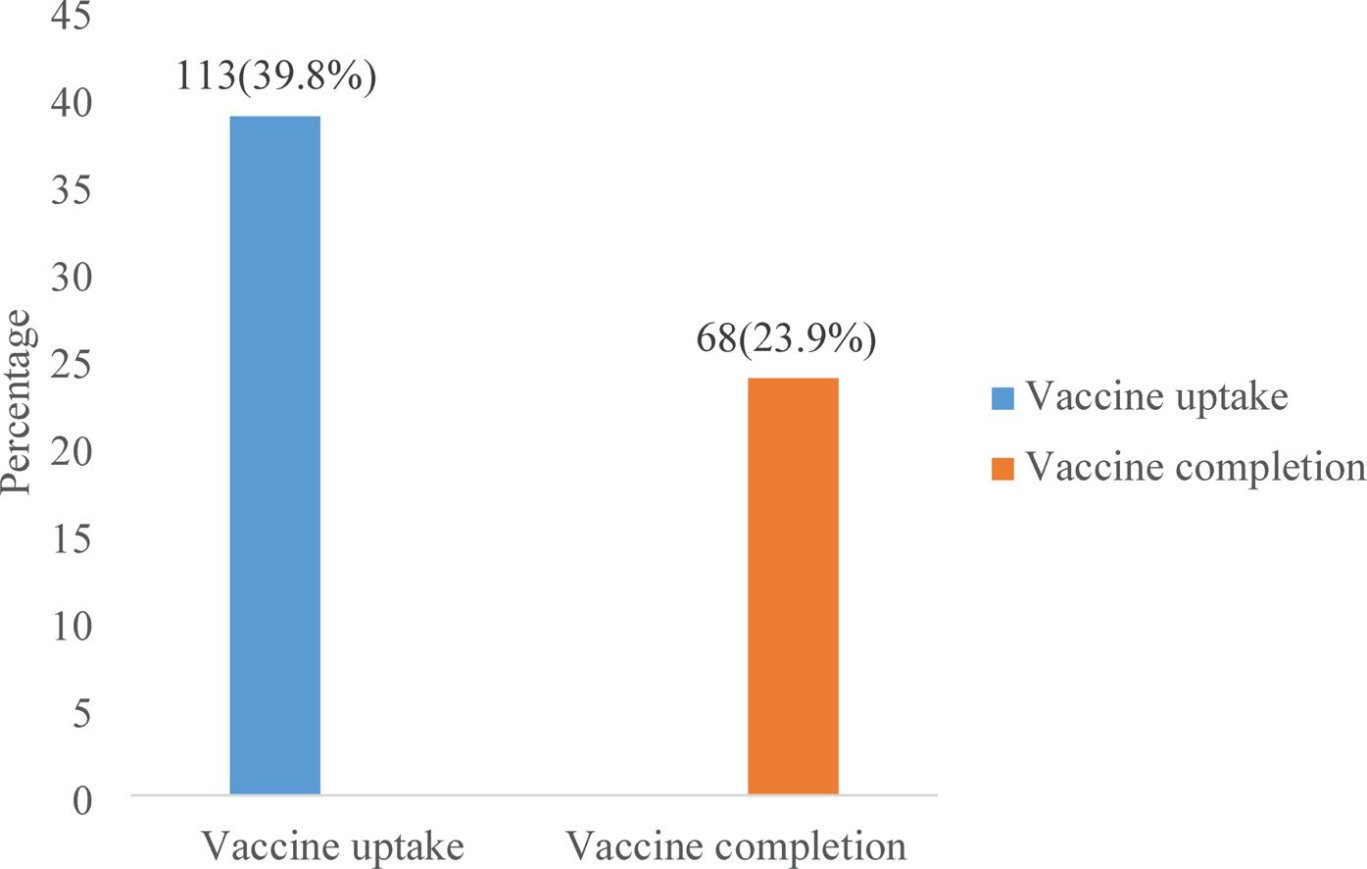
Prevalence of hepatitis B vaccine uptake and completion

Out of 284 participants, 113 (39.8%) reported having received at least one dose of the hepatitis B vaccine. Regarding completion, only 68 (23.9%) participants had received the full three doses of the vaccine (Fig. 1).

### Factors associated with uptake of hepatitis B vaccination

The study revealed that the odds of reporting uptake of hepatitis B vaccine among participants was significantly associated with year of study, program level, knowledge of hepatitis B, and awareness of mass vaccination campaigns. Students in Year 2 (AOR = 4.2, *p* = 0.002), Year 3 (AOR = 1.5, *p* < 0.001), and Year 4 (AOR = 1.69, *p* = 0.010) were more likely to report uptake of hepatitis B vaccine when compared to those in Year 1. Additionally, bachelor’s degree students (AOR = 0.32, *p* = 0.008) were more likely to report being vaccinated than diploma students. Participants who were knowledgeable about hepatitis B (AOR = 2.34, *p* = 0.001) and those aware of mass vaccination campaigns (AOR = 0.54, *p* = 0.03) demonstrated significantly higher vaccination rates. However, factors such as age, sex, marital status, religion, and training attendance did not show significant associations with vaccination uptake in the multivariate analysis (Table 2).

**Table 2:** Bivariate and multivariable analysis of factors associated with uptake of Hepatitis B vaccine (*n* = 284)

Variable	Ever vaccinated		COR (95% CI)	*P*-Value	AOR (95% CI)	*P*-Value
	Yes *n* (%)	No *n* (%)				
**Age**						
< 20	11(20.8)	42(79.2)	1			
21–31	93(41.9)	129(58.1)	0.5(0.12–1.23)	0.21		
> 32	9(100)	0(0.00)	1.2(0.01–2.30)	0.99		
**Sex**						
Male	37(33.9)	72(66.1)	1			
Female	76(43.4)	99(56.6)	0.67(0.41–1.10)	0.11		
**Marital status**						
Single	99(37.9)	162(62.1)	1			
Married	12(63.2)	7(36.8)	1.64(0.23–11.8)	0.63		
Other	2(50.0)	2(50.0)	0.58(0.067–5.11)	1.61		
**Year of study**						
Year 1	13(19.4)	54(80.6)	1		1	
Year 2	24(28.6)	60(71.4)	8.1(3.31–19.41)	**< 0.001**	4.2(1.23–3.45)	**0.002**
Year 3	49(53.3)	43(46.7)	4.82(2.17–10.74)	**< 0.001**	1.5(1.12-2.00)	**< 0.001**
Year 4	27(65.9)	14(34.1)	1.69(0.79–3.64)	**0.177**	1.69 (1.37–2.09)	**0.010**
**Program level**						
Diploma	32(28.6)	80(71.4)	1		1	
Bachelor	81(47.1)	91(52.9)	0.45(0.27–0.75)	**0.002**	0.32(0.43–0.76)	**0.008**
**Religion**						
Christian	83(40.7)	121(59.3)	1			
Muslim	30(40.5)	44(59.5)	0.52(0.23–2.43)	0.32		
Other	0(0.00)	6(100)	0.80(0.2–1.34)	0.61		
**Received training on Hepatitis infection and prevention (** *n* ** = 283)**						
No	28(19.2)	118(80.8)	1		1	
Yes	85(62.0)	52(38.0)	6.88(1.23–8.80)	**0.002**	1.00 (0.60–2.50)	0.410
**Knowledge of Hepatitis B**						
Not knowledgeable	11(21.6)	35(78.4)	1		1	
Knowledgeable	102(47.7)	126(55.3)	1.56 (1.57–3.09)	**< 0.001**	2.34(1.20–4.81)	**0.001**
**Perceived Hepatitis B vaccine effectiveness**						
No	28(27.7)	73(72.2)	1		1	
Yes	85(47.8)	93(52.2)	2.54(1.50–6.20)	**0.004**	1.00 (0.45–2.10)	0.320
**Awareness of mass vaccination**						
No	30(20.2)	116(79.8)	1		1	
Yes	87(64.0)	50(36.0)	0.77(0.62–0.96)	**0.021**	0.54(0.62-0.087)	**0.03**

COR = crude odds ratio; AOR = adjusted odds ratio, Bolded=significant, *p* < 0.05

### Factors associated with completion of hepatitis B vaccination

The study identified several factors significantly associated with completion of hepatitis B vaccination. Participants in Bachelor programs (AOR = 1.8, *p* = 0.001) were more likely to complete vaccination than Diploma students. Knowledgeable individuals on hepatitis infection (AOR = 5.34, *p* = 0.001) and those who received training (COR = 3.48, *p* = 0.001) had significantly higher completion rates. Additionally, participants who perceived the vaccine as effective (COR = 3.54, *p* = 0.03) and those aware of mass vaccination campaigns (AOR = 0.22, *p* = 0.001) were more likely to complete the series. Completion rates were higher in students from higher years of study, though not statistically significant. Age, sex, marital status, and religion showed no significant association with vaccine completion (Table 3).

**Table 3:** Bivariate and multivariable analysis of factors associated with completion of hepatitis B vaccine (*n* = 284)

Variable	Completion of vaccination		COR (95% CI)	*P*-Value	AOR (95% CI)	*P*-Value
	Yes *n* (%)	No *n* (%)				
**Age**						
< 20	9(16.8)	44(83.2)	1			
21–31	90(39.7)	131(60.3)	0.47(0.08–2.65)	0.98		
> 32	6(61.9)	3(38.1)	1.95(0.68–5.56)	0.30		
**Sex**						
Male	35(31.9)	74(68.1)	1			
Female	70(39.6)	105(60.4)	2.81(0.04–7.52)	0.41		
**Marital status**						
Single	90(35.7)	171(64.3)	1			
Married	10(59.6)	9(40.4)	1.20(0.37–3.83)	0.56		
Other	0(0.0)	4(100.0)	2.27(0.99–5.20)	0.21		
**Year of study**						
Year 1	10(14.1)	57(85.9)	1			
Year 2	20(24.8)	64(75.2)	0.88(0.42–1.85)	1.3		
Year 3	43(50.8)	46(49.2)	1.18(0.58–2.41)	0.54		
Year 4	27(62.8)	14(37.2)	1.58(0.78–3.21)	0.87		
**Program level**						
Diploma	30(26.9)	82(73.1)	1			
Bachelor	79(45.9)	93(54.1)	0.95(1.55–1.65)	**0.03**	1.8 (0.09–0.57)	**0.001**
**Religion**						
Christian	80(38.7)	124(61.3)	1			
Muslim	27(39.8)	47(60.2)	0.66(0.34–1.28)	0.35		
Other	0(0.00)	6(100)	1.16(0.70–1.93)	0.10		
**Received training on Hepatitis infection and prevention (** *n* ** = 283)**						
No	22(17.9)	121(82.1)	1			
Yes	80(59.4)	57(40.6)	3.48(2.20–8.90)	**0.001**	2.3(1.2–7.6)	**0.021**
**Knowledge of Hepatitis B**						
Not knowledgeable	10(19.6)	36(80.4)	1		1	
Knowledgeable	100(41.7)	128(58.3)	1.33 (1.47–5.09)	**< 0.001**	5.34(2.20–4.71)	**0.001**
**Perceived Hepatitis B vaccine effectiveness**						
No	24(24.8)	77(75.2)	1		1	
Yes	80(46.9)	98(53.1)	3.54(0.30–1.20)	0.03	1.48 (0.40–5.20)	0.420
**Awareness of mass vaccination**						
No	80(68.0)	57(32.0)	1		1	
Yes	8(19.8)	43(80.2)	0.2(0.12–0.36)	**< 0.001**	0.22(0.12-0.027)	**0.001**

COR = crude odds ratio; AOR = adjusted odds ratio, Bolded=significant, *p* < 0.05

### Participants’ perspectives on hepatitis B vaccine uptake and completion

The results of Key Informant Interviews (KIIs) with students revealed several key themes regarding hepatitis B vaccine uptake and completion. The qualitative findings were organized into five main themes and corresponding subthemes reflecting participants’ perspectives on vaccination behavior among university students. These themes included knowledge and awareness of hepatitis B and the vaccine, perceived susceptibility and risk, accessibility of vaccination services, influence of peers and family, and motivators and barriers to vaccine completion. The identified themes and corresponding subthemes are summarized in Table 4.

**Table 4:** Themes and subthemes on participants’ perspectives on hepatitis B vaccine uptake and completion

Main Theme	Subthemes	Illustrative Meaning from Participants
Knowledge and Awareness of Hepatitis B and the Vaccine	• Limited knowledge on Hepatitis B transmission and consequences • Misconceptions about vaccine safety and efficacy • Lack of knowledge on number of vaccine doses	Many students were aware of the vaccine but lacked clear knowledge about its safety, effectiveness, and required number of doses, leading to incomplete vaccination.
Perceived Susceptibility and Risk	• High perceived risk due to clinical exposure • Low perceived personal risk leading to vaccine hesitancy	Students who perceived themselves at higher risk were more motivated to initiate and complete vaccination, while those who felt at low risk delayed vaccination.
Accessibility and Convenience of Vaccination Services	• Limited vaccine availability • Inconvenient clinic hours • Long waiting times • Unclear vaccination schedules	Logistical barriers such as availability, time constraints, and unclear schedules contributed to missed or incomplete doses.
Influence of Peers and Family	• Positive encouragement from peers • Negative perceptions from family members	Social influence from friends and family played an important role in motivating or discouraging vaccination uptake.
Motivators and Barriers to Completion	• Institutional requirements (clinical placement) • Health awareness and campaigns • Forgetfulness and academic workload • Fear of side effects	Institutional requirements and awareness promoted completion, while fear, forgetfulness, and competing academic priorities delayed follow-up doses.

Each theme is described in detail below with supporting quotations from participants to illustrate their experiences and perceptions regarding hepatitis B vaccine uptake and completion.

### Theme 1: Knowledge and awareness of hepatitis B and the vaccine

Many students demonstrated limited knowledge about Hepatitis B transmission, its health consequences, and the benefits of vaccination. Some participants were aware of the vaccine but held misconceptions about its safety and efficacy.

*I heard about the Hepatitis B vaccine*,* but I am not sure if it is completely safe. I don’t want to get sick from it.* (P01, Male, 21 years)

The participant indicating knowledge gap on vaccine, vaccination and possible side effects of the vaccine. This influenced him of not opting to get vaccination as his misconception on health-related consequences as a result of getting vaccinated was not cleared with appropriate knowledge.

Some participants were aware of the existence of Hepatitis B vaccine but had knowledge gap on the number of doses to be taken for complete vaccination. This is evidenced by one participant who narrated;

*I know there is a vaccine*,* but I don’t know how many doses we are supposed to take.* (P02, Female, 20 years)

In such instances of limited awareness, a number of participants reported to likely adopt one dose and not follow up for the next doses in case such knowledge was not provided by the health care provider. Some of them even considered adopting single dose vaccine to offer full protection in a similar manner as the full vaccination.

### Theme 2: Perceived susceptibility and risk

Students perceived risk influenced vaccination decisions. Those who considered themselves at higher risk due to clinical exposure, sexual activity, or family history were more likely to get vaccinated, while others who perceived low risk delayed or avoided vaccination. This was evidenced by the following narration from a participant who opted for vaccine;

*Since I sometimes help at the hospital during practicals*,* I thought it would be safer to get the vaccine.* (P03, Female, 22 years)

Individual perceived risk of exposure to Hepatitis B virus was narrated to a very strong factor for completion of vaccination. This 22-year-old female indicated that she was intrinsically motivated to be safe and vowed to ensure that all doses are taken for her to be safe. She was also motivated to seek more information about vaccine and made sure her knowledge of vaccine was good.

While perceived higher risk of hepatitis B exposure motivated positive health seeking behavior, the reverse also existed. Those who perceived at low risk of exposure to hepatitis B narrated to adopt low attention on the vaccine, and eluded it to be for those who are at higher risk of exposure. This is evidenced by the following quotation from a participant who studied courses that do not involve with invasive procedures with clients, and considered himself careful with his lifestyle;

*I don’t think I need it because I am careful and don’t take unnecessary risks.* (P04, Male, 19 years)

### Theme 3: Accessibility and convenience of vaccination services

Limited availability of vaccines on campus and associated costs were frequently cited barriers. These logistical challenges often led to incomplete vaccination. Often students spend more time at the University fully occupied with studies, and only limited time is available for other activities. Usually, vaccine has to be taken from the hospital, which presents other geographical challenge as narrated by one of the participant;

*The vaccine is not always available at the university*,* and sometimes I have to go to Hospital to get it. That makes it difficult to complete all doses. (*P05, Male, 23 years*)*

Timing was also described to be another challenge by participants. While hospital clinics are always open during working hours, it is the same hours when students are supposed to be in class or field for studies. Often free time for students are during early morning and late evening hours, where such time clinics to offer vaccine are closed. This was backed by narration from one of the participant interviewed who described her personal challenge as;

*Sometimes I plan to go for the next dose*,* but the clinic is closed or no phone call or any reminder. (*P06, Female, 21 years*)*

Some participants who opted to use free time mid-class sessions or take time-off from studies to rush for the dose during their appointment days experienced a health system challenge. Waiting time at facility, availability of a health provider at all time or availability of vaccine failed some participants who rushed to the facility to take the vaccine such that they had to opt for “getting back to class rather than wait for vaccine” as described below by one of the participant;

*Sometimes the person providing the vaccine is not available*,* or it is time for my classes*,* so I have to miss the dose. (*P07, Female, 22 years*)*

Sometimes personal miss-understanding of their schedule for vaccination do affect adoption of subsequent doses of vaccination. While this may seem to be an individual problem, it affected adoption of remaining doses of vaccine. While second and third dosing was easily explained to be after one month and six months from the first dose respectively, without indicating exact dates for adopting such vaccines created a challenge to some participants. Some described this as timetable for subsequent dosing being not clear as per participant below, and in some challenges to recall sets in;

*Sometimes the timetable for taking the doses is not clear*,* so I’m not sure when to come for the next one. (*P08, Female, 22 years*)*

### Theme 4: Influence of peers and family

Peer and family opinions significantly influenced students’ decisions. Positive encouragement motivated vaccine uptake, while skepticism or negative attitudes discouraged some participants. Commenting on positive influence received from friends who were considered to be significant others for the participant, a 21-year-old female participant who managed to complete all vaccine doses commented;

*My friend told me it is important to get all three doses*,* so I made sure to complete them.* (P09, Female, 21 years)

On the other hand, one participant indicated to have received negative comment from a brother with regard to the importance of hepatitis B vaccine. The participant narrated;

*My brother said vaccines are not that important*,* so I didn’t bother at first.* (P10, Male, 20 years)

However, during discussions with friends and colleagues at the college, he came to realise that the vaccine was very important to him and its protective value superseded the reasons provided earlier by his brother. He then considered the brother to have inadequate knowledge of the hepatitis B vaccine and decided to overrule the advice and received the vaccine.

### Theme 5: Motivators and barriers to completion

Key motivators included health consciousness, exposure to health campaigns, and university program requirements. Most medical and nursing students were motivated by university program requirement to complete the dose to allow them participate well in the clinical sessions. One of the participants who completed the vaccine doses provided below narration as the main reason that made her adhere to vaccine schedule to completion;

*I completed the series because it is required before starting clinical attachments.* (P11, Female, 23 years)

Barriers included forgetfulness, competing academic priorities, and fear of side effects. The two participants who failed to adhere to dose schedules narrated;

*I wanted to finish the doses*,* but I kept forgetting due to exams and assignments.* (P12, Male, 22 years). And the other also reported that; *I was afraid the next dose would make me feel sick*,* so I delayed it.* (P13, Male, 21 years)

## Discussion

The findings of this study indicate suboptimal hepatitis B vaccine uptake and completion among university students, which is consistent with previous studies conducted among university populations in low- and middle-income countries. Similar low vaccination uptake has been reported in Uganda and Nigeria, where inadequate awareness and limited access to vaccination services were identified as key barriers to full immunization [5, 11].Likewise, studies in Ethiopia have shown that incomplete hepatitis B vaccination schedules are common among health science students, largely due to lack of knowledge, misconceptions about vaccine safety, and competing academic demands [18]. These findings align with the current study where knowledge and awareness were significant predictors of vaccine uptake and completion.

In the present study, 23.9% of participants completed all three doses of the hepatitis B vaccine, which is higher than the 5.8% completion rate reported among students at Wolkite University in Ethiopia [18]. The higher completion rate in this study may reflect better awareness and access to vaccination services among students. However, the completion rate remains below the level required for adequate protection among high-risk groups. Strengthening vaccination programs, improving awareness, and ensuring vaccine availability within universities are essential to increase completion rates [5, 11].Similarly, a study in Uganda reported that only 26.7% of adults had received all three doses, and those educated about the vaccine were significantly more likely to complete vaccination (AOR = 4.39) [19]. Furthermore, studies among healthcare students in Vietnam demonstrated that 89.8% received at least one dose however, only 63.3% completed all three doses [20]. These findings indicate that even in populations with high awareness, completion rates remain suboptimal. Additionally, a meta-analysis of 35 studies across sub-Saharan Africa found that the average completion rate was only 24.7% (95% CI: 17.3–32.0), highlighting that low completion is a widespread issue rather than an isolated problem [21].

Several factors were found to hinder completion of the hepatitis B vaccination series, consistent with challenges reported in previous studies. In this study, limited vaccine availability was a significant barrier, reflecting similar observations among healthcare workers in Sudan, where unavailability of vaccines at health facilities prevented many from completing the full series [22]. Similarly in Kenya, students identified unavailability (35.8%) and timing issues (29.4%) as primary obstacles [23]. Financial constraints also contributed to incomplete vaccination. Participants in the current study who could not afford the cost of additional doses often failed to complete the series, a trend reported in other African settings such as Nigeria and Kenya, where out-of-pocket expenses for vaccines were associated with lower completion rates among students and healthcare workers [21, 23]. Additionally, in Somali students cited cost, lack of knowledge, and side-effect fears [24].

Inconvenient scheduling and competing commitments limited adherence in the present study, consistent with findings from Vietnam and Ghana, where busy academic and work schedules were major reasons for missing second or third doses [20, 25]. Knowledge gaps were also observed; some participants were unaware of the importance of completing all three doses or misunderstood the timing between doses. Similar gaps were reported among nursing students in Greece and Uganda, where low awareness of the vaccination schedule contributed to incomplete coverage [26, 27].

Personal attitudes and beliefs, such as fear of side effects and low perceived susceptibility, reduced completion in this study, echoing findings from Somalia and Kenya, where apprehension about vaccine safety and doubts about efficacy were commonly cited as barriers [23, 24]. Overall, these comparisons suggest that the barriers observed in the current population reflect a broader, well-documented pattern across diverse African and international settings, emphasizing the need for targeted strategies to address structural, educational, and behavioral factors that impede vaccine completion.

Improving strategies to ensure availability and affordability of Hepatitis B vaccine and vaccination have demonstrated improved adoption and completion. On-campus vaccination clinics and subsidized or mandatory vaccination programs have enhanced completion in other settings [26, 28]. For instance, Ghanaian healthcare workers who received training and perceived higher risk demonstrated significantly better completion rates [25]. Tailoring interventions to students in lower academic years and diploma programs could address coverage gaps identified in our population.

Globally, hepatitis B vaccine completion among students remains low. In Greece, only 27.7% of nursing students were fully vaccinated, and completion correlated with year of study and knowledge level [26]. In Uganda, completion of the hepatitis B vaccination series among nursing and health-related students remains poor, reflecting trends reported across East African student populations [5, 27] These results reinforce the trend that even in regions with high vaccine awareness, full compliance remains unsatisfactory [5].

### Strengths and limitation of the study

This study has several strengths, including its focus among college students in a medical university, studying a high risk and understudied population on hepatitis B exposure in terms of sexual activity and potential hazards resulting from clinical interaction with clients, blood and bodily fluids during clinical training and services. The use of both quantitative and qualitative methods enabled a comprehensive understanding of contextual factors and experiences that influenced uptake of vaccine. Results of this study may need to be interpreted with caution. Firstly, the intrinsic weakness of a cross-sectional study design that it cannot establish a causal relationship. Secondly, that we assumed all participants required hepatitis B vaccination and did not verify their childhood immunization history. Tanzania through the Expanded Program for Immunization (EPI) introduced Hepatitis B vaccine into the routine childhood immunization schedule in early 2000s, and have been administered as part of the other vaccines (together with Diphtheria, Pertussis and Tetanus) at 6, 10, and 14 weeks of age (*Ministry of Health and Social Welfare (Tanzania).* Expanded Programme on Immunization: Immunization and Vaccine Development in Tanzania. *Dar es Salaam: Ministry of Health and Social Welfare; 2008.*). Since our study population was aged 17 years and above, a proportion of participants—especially those aged 17–23 years—may have received the full primary vaccination during childhood following its national introduction, and booster doses is not routinely recommended by WHO (*World Health Organization.* Hepatitis B vaccines: WHO position paper – July 2017. *Geneva: WHO; 2017*). While the vaccination status was based on self-report and researcher didn’t review childhood records, some participants may have been misclassified as unvaccinated and therefore under estimating the proportion of vaccine uptake.

## Conclusion

This study highlights hepatitis B vaccine uptake with low completion of the full three-dose among participants. Key factors influencing vaccination completion included program level, knowledge of hepatitis B, training attendance, and awareness of mass vaccination campaigns. These findings align with similar studies in Africa and globally, which consistently show that while many initiate vaccinations, few complete it. To improve coverage, targeted educational interventions, accessible vaccination services, and reminder systems are essential. Strengthening institutional support and raising awareness on the importance of full vaccination will be crucial in achieving higher completion rates and long-term protection.

## Supplementary Information

Below is the link to the electronic supplementary material.

Supplementary Material 1

Supplementary Material 2

## Data Availability

The datasets used during the current study are available from the corresponding author on request and approval from the KCMC University.
