# Supplementary material for: Hepatitis B vaccine uptake, completion, and associated factors among university students in Tanzania: a mixed method study at KCMC University, Moshi, Tanzania
Source: BMC Infect Dis. 2026 May 4;26:1187. doi: 10.1186/s12879-026-13501-5 (PMC13289535; doi:10.1186/s12879-026-13501-5)
Supplement: Supplementary file 1 — Supplementary Material 1 [file 12879_2026_13501_MOESM1_ESM.docx]

**Supplementary File 1. English Version of the Data Collection Tool (Questionnaire)**

**Study Title:** Uptake and Completion of Hepatitis B Vaccination Among Diploma and Undergraduate Students at KCMC University, Moshi, Tanzania

**Instructions:** Please answer all questions honestly. Your responses will be kept confidential.

**Section A: Socio-Demographic Information**

1. Age: ______
2. Sex: ☐ Male ☐ Female ☐ Other
3. Program level: ☐ Diploma ☐ Undergraduate
4. Faculty/Programme: __________________
5. Year of study: ☐ 1 ☐ 2 ☐ 3 ☐ 4 ☐ 5
6. Marital status: ☐ Single ☐ Married ☐ Other
7. Religion: ☐ Christian ☐ Muslim ☐ Other

**Section B: Exposure, Training and Risk Factors**

1. Have you ever been exposed to blood or body fluids of others? ☐ Yes ☐ No
2. Do you have any medical conditions that affect immunity? ☐ Yes ☐ No
3. Have you ever attended any training or health education session on Hepatitis infection and prevention? ☐ Yes ☐ No

**Section C: Awareness**

1. Have you ever heard about Hepatitis B infection? ☐ Yes ☐ No
2. Are you aware of any mass Hepatitis B vaccination campaigns conducted in Tanzania? ☐ Yes ☐ No ☐ Not sure

**Section D: Knowledge of Hepatitis B**

1. Hepatitis B can be transmitted through:
   ☐ Blood transfusion
   ☐ Unprotected sexual contact
   ☐ Sharing needles
   ☐ Contaminated food/water
   ☐ Mother to child
2. Hepatitis B can be prevented by:
   ☐ Vaccination
   ☐ Using condoms
   ☐ Avoiding sharing sharp objects
   ☐ Healthy diet
3. Hepatitis B infection can be treated: ☐ Yes ☐ No ☐ Not sure
4. Hepatitis B can lead to serious complications such as liver cirrhosis or liver cancer. ☐ Yes ☐ No ☐ Not sure

**Section E: Vaccination Status**

1. Have you taken the Hepatitis B vaccine? ☐ Yes ☐ No
2. If yes, how many doses have you received?
   ☐ 1 ☐ 2 ☐ 3
3. If not vaccinated, what is the main reason? __________________________

**Section F: Perceptions on Uptake and Completion of Hepatitis B Vaccination**

Rate your agreement with the following statements
(1 = Strongly Disagree, 5 = Strongly Agree)

**Perceived Susceptibility**
20. I am at risk of contracting Hepatitis B. ☐1 ☐2 ☐3 ☐4 ☐5

**Perceived Severity**
21. Hepatitis B is a serious health problem. ☐1 ☐2 ☐3 ☐4 ☐5

**Perceived Benefits**
22. Vaccination can protect me from Hepatitis B. ☐1 ☐2 ☐3 ☐4 ☐5

**Perceived Vaccine Effectiveness**
23. The Hepatitis B vaccine is effective in preventing infection. ☐1 ☐2 ☐3 ☐4 ☐5

**Perceived Barriers**
24. Getting vaccinated is inconvenient or expensive. ☐1 ☐2 ☐3 ☐4 ☐5

**Self-Efficacy**
25. I am confident I can complete the full three-dose vaccination schedule. ☐1 ☐2 ☐3 ☐4 ☐5

**Cues to Action**
26. Reminders from health services or peers will encourage me to get vaccinated. ☐1 ☐2 ☐3 ☐4 ☐5

**Section G: Additional Practices**

1. Do you practice any measures to prevent Hepatitis B (e.g., avoiding sharing needles, safe sex)? ☐ Yes ☐ No
2. If yes, please specify: __________________________
