# Supplementary material for: Hepatitis B vaccine uptake, completion, and associated factors among university students in Tanzania: a mixed method study at KCMC University, Moshi, Tanzania
Source: BMC Infect Dis. 2026 May 4;26:1187. doi: 10.1186/s12879-026-13501-5 (PMC13289535; doi:10.1186/s12879-026-13501-5)
Supplement: Supplementary file 2 — Supplementary Material 2 [file 12879_2026_13501_MOESM2_ESM.docx]

**Supplementary File 2. Semi-Structured Key Informant Interview Guide (KCMC University)**

**Study Title:** Motivators and Barriers to Hepatitis B Vaccination Uptake and Completion Among Diploma and Undergraduate Students at KCMC University, Moshi, Tanzania

**Instructions:**

- Explain purpose of interview and obtain verbal consent.
- Inform participants that responses are confidential and participation is voluntary.
- Encourage detailed responses with probing questions (e.g., “Can you explain further?”, “Can you give an example?”).

**Theme 1: Knowledge and Awareness of Hepatitis B and the Vaccine**

1. How familiar are students with Hepatitis B infection and its complications?
2. What do students know about the Hepatitis B vaccine?
3. Are there common misconceptions about Hepatitis B or the vaccine?
4. How do students usually learn about Hepatitis B (e.g., lectures, campaigns, peers, media)?

**Theme 2: Perceived Susceptibility and Risk**

1. Do students perceive themselves at risk of contracting Hepatitis B? Why or why not?
2. Are some groups of students perceived to be at higher risk?
3. How does perceived risk influence students’ decisions to get vaccinated?

**Theme 3: Motivators and Barriers to Completion**

1. What factors motivate students to complete the full three-dose Hepatitis B vaccination schedule?
2. What challenges or barriers prevent students from completing the vaccine schedule?
   - Cost
   - Side effects
   - Forgetfulness
   - Lack of information

**Theme 4: Influence of Peers and Family**

1. How do peers influence students’ vaccination decisions?
2. Does family support or advice affect vaccine uptake?
3. Can you provide examples of how social networks encourage or discourage vaccination?

**Theme 5: Accessibility and Convenience of Vaccination Services**

1. How accessible are vaccination services for students (location, timing, availability)?
2. Are there logistical issues that make vaccination inconvenient?
3. What improvements could make vaccination services more user-friendly?

**Theme 6: Recommendations**

1. What strategies would you suggest to improve awareness, uptake, and completion of Hepatitis B vaccination among students?
